# Pseudoaneurysm of the ascending aorta: case report of a donor-derived Pseudomonas infection in a heart transplant recipient

**DOI:** 10.1186/s12879-021-06557-y

**Published:** 2021-08-21

**Authors:** Szilvia Kugler, Miklós Pólos, Ákos Király, Ákos Pataki, Ádám Koppányi, Tamás Varga, Zsófia Szakál-Tóth, Nóra Parázs, Tímea Teszák, Zoltán Tarjányi, Gyula Prinz, István Hartyánszky, Zoltán Szabolcs, Béla Merkely, Balázs Sax

**Affiliations:** 1 Heart and Vascular Center, Semmelweis University, 68 Városmajor Street, 1122 Budapest, Hungary; 2 Department of Anesthesiology and Intensive Therapy, Semmelweis University, 68 Városmajor Street, 1122 Budapest, Hungary

**Keywords:** Aortic pseudoaneurysm, *Pseudomonas aeruginosa*, Heart transplantation, Donor-derived infection, Case report

## Abstract

**Background:**

Mycotic aortic pseudoaneurysm is a rare complication after heart transplantation (HTX) with remarkable mortality. Intrathoracic infection is a well-documented predisposing factor for this disease. *Staphylococcus aureus*, *Pseudomonas aeruginosa* or Candida species are commonly isolated from resected specimens of the pseudoaneurysms. We demonstrate a unique case of mycotic pseudoaneurysm caused by presumably donor-derived Pseudomonas infection in a heart transplant recipient.

**Case presentation:**

Our 67-year-old male patient treated with diabetes mellitus underwent HTX. The donor suffered from epiglottic abscess and pneumonia with known microorganisms including Pseudomonas, therefore both the donor and recipient received targeted antimicrobial therapy and prophylaxis. Five months after the uneventful HTX, lab test of the asymptomatic patient showed moderate, increasing C-reactive protein level without obviuos source of infection. Chest computed tomography showed a large (90 mm) saccular dilatation of the tubular portion of ascending aorta. Urgent surgical intervention identified a pseudoaneurysm, histological examinations and cultures of the resected aorta verified *Pseudomonas aeruginosa* aortitis, while all blood cultures remained negative. Retrospective interrogation of other transplanted organs of the donor supported donor-derived infection as the transport fluid of the right kidney grew Pseudomonas. The patient received 3 weeks of ceftazidime followed by 7 months of oral ciprofloxacin therapy. One year after the operation the patient was asymptomatic with normal inflammatory markers.

**Conclusions:**

Donor-derived infection is a rare but potential cause of aortitis. Early diagnosis, surgical intervention and adjuvant antibiotic therapy seem to be the keys to successful management of mycotic pseudoaneurysms after HTX.

## Background

After open heart surgeries, pseudoaneurysms of the thoracic aorta may develop from cannulation sites, suture lines or anastomotic sites [1]. Microorganisms tend to localize at the sites of previous injury in the wall of the vessel which predisposes to the formation of infected (or so called mycotic) pseudoaneurysms [2]. After heart transplantation (HTX), immunosuppression renders patients vulnerable to infection, which may lead to increased risk for aortic suture line insufficiency and the formation of mycotic pseudoaneurysms compared to immunocompetent individuals. Resected specimen of the mycotic ascending aortic pseudoaneurysms of heart transplantat patients frequently grows *Staphylococcus aureus*, Pseudomonas (P.) aeruginosa or Candida species. Based on literature data discussed in details below, two-thirds of patients recover after treatment. Despite the potentially fatal outcome of these complications, there are some controversial issues regarding the optimal surgical and antibiotic treatment.

We describe the case of an almost asymptomatic patient in whom a *P. aeruginosa* pseudoaneurysm of the ascending aorta developed five months after HTX at the aortic suture line. In this case no obvious concomitant infection was found at the patient and donor-derived infection is suspected. To the best of our knowledge, this is the first case, in which a mycotic aortic pseudoaneurysm was formed due to a probably donor-derived *P. aeruginosa* infection. We demonstrate the diagnostic process, discuss the antibiotic management and surgical treatment and also review the relevant literature.

## Case presentation

### Patient information

A 67-year-old man who had undergone orthotopic HTX 5 months before was admitted to our clinic for a surveillance endomyocardial biopsy (EMB) in perfect physical condition and asymptomatic. There was no history of fever episodes, other symptoms of infection, dyspnea, chest pain or malaise.

His medical history is significant for hypertension and chronic renal failure, the etiology of end stage heart failure was dilated cardiomyopathy with non-significant coronary stenosis. Brain death of the 31-year-old donor developed after a successful reanimation from a respiratory and cardiac arrest which was caused by an epiglottic abscess. The donor was septic with fever and high procalcitonin level (maximum 16 ng/ml). Cultures of the abscess grew Klebsiella aerogenes, Enterococcus faecalis, *Staphylococcus aureus*, Hafnia alvei, Finegoldia magna, Prevotella bivia and Prevotella oris. Chest x-ray and ultrasound showed right sided basal pneumonia. Microbiological examination of bronchoalveolar lavage fluid revealed the presence of Candida kefyr and multi-sensitive *P. aeruginosa* (results were validated on the day after transplantation). Donor received initial ceftriaxone therapy for 6 days (2000 mg once daily) that was changed to imipenem/cilastatin (500 mg four times daily), given for the last 24 h before donation.

Immunosuppression of the patient consisted induction therapy (3-day course of antithymocyte globulin and high-dose methylprednisolon) and triple maintenance immunosuppressive regimen including methylprednisolone (8 mg once daily), tacrolimus (aiming a trough level of 10–15 ng/ml in the first 3 months, then 8–10 ng/ml) and mycophenolic acid (720 mg twice daily). Perioperative prophylaxis considering the microbiology culture results of donor consisted of meropenem (1000 mg three times daily) and vancomycin (1000 mg twice daily), given for 48 h. The postoperative course was uncomplicated. Transthoracic echocardiographies (TTE) revealed good graft function with normal estimated pulmonary artery systolic pressure. No rejection episodes could be found with surveillance endomyocardial biopsies.

Retrospectively, outcomes of other transplanted organs were interrogated from Eurotransplant. Liver, both kidneys, vascular homografts and connective tissue were explanted from the same donor. Transport fluid of the right kidney grew Pseudomonas, therefore the recipient of this organ received piperacillin/tazobactam prophylaxis. Preservation fluids of other organs were not cultured. Neither the recipient of the left kidney, nor liver recipient received any anti-Pseudomonas prophylaxis. No donor-derived infection was reported after transplantation of left and right kidneys, liver and connective tissue. Vascular homografts were discarded for safety reasons.

### Clinical findings

At the beginning of June, the patient was scheduled for a regular EMB procedure. He presented with no symptoms except of a gradual weight loss of 15 kg (19% of his initial body weight) during 4 months. Physical examination was unrevealing. His blood pressure had been in target range for several weeks. No fever or subfebrility was detected.

### Diagnostic assessment

Routine lab tests showed normal white blood cell count (8,9 G/l) with 87% neutrophyl granulocytes and a remarkable C-reactive protein (CRP) elevation showing an increasing tendency in the previous two months (7–24–31–57 mg/l). In addition, normocyter anemia (hemoglobin 104 g/l, hematocrit 33%) and impaired kidney function (glomerular filtration rate 39 ml/min, creatinin 155 µmol/l) was detected. Urinanalysis excluded pyuria or bacteruria. SARS-CoV-2 polymerase chain reaction test was negative.

**Fig. 1:**
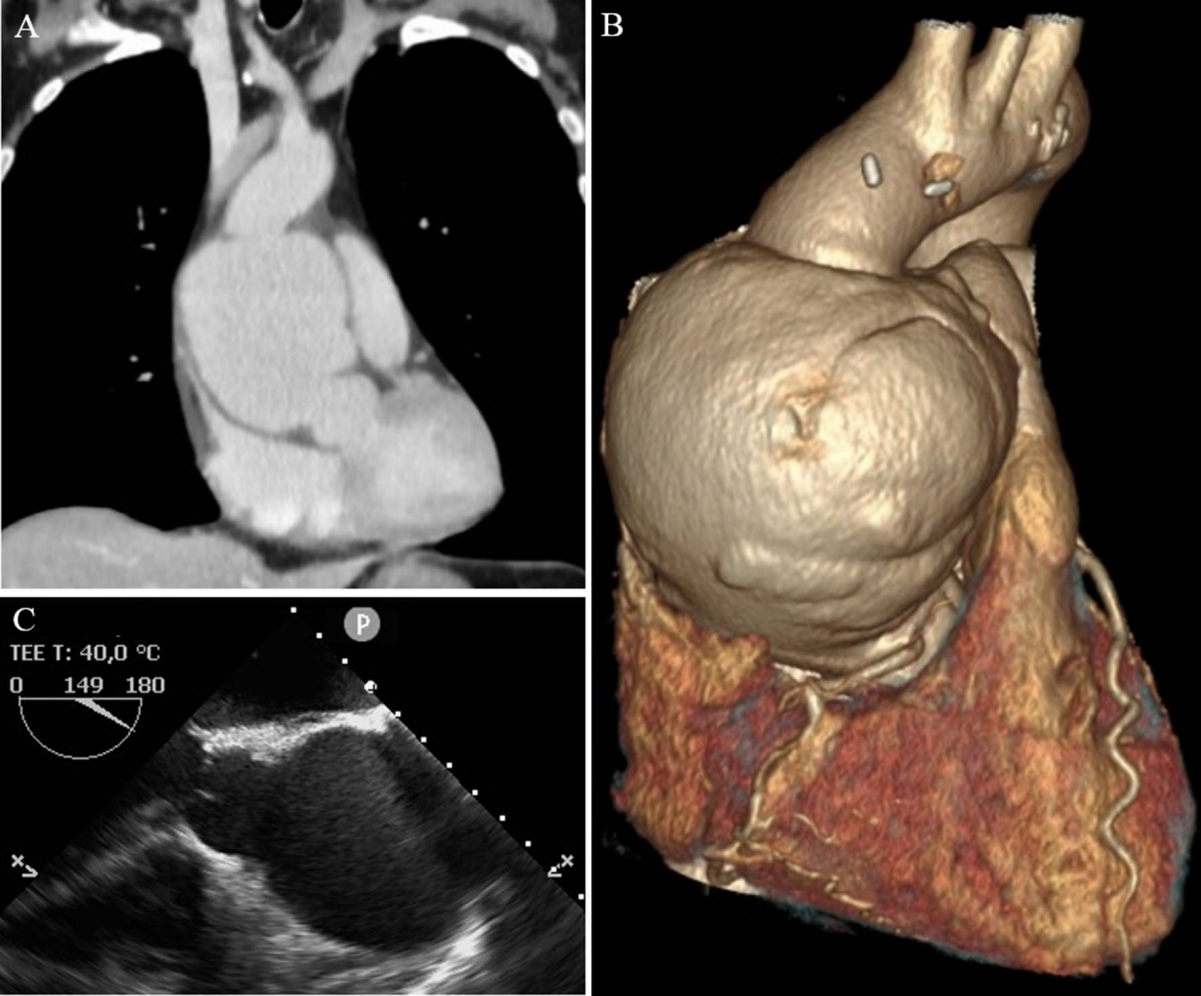
Perioperative imaging of the ascending aortic pseudoaneurysm. Coronal plane of CT scan (**A**) and three-dimensional CT reconstruction (**B**) showed a huge, saccular dilatation of the tubular portion of ascending aorta. This abnormality could be also visualised by intraoperative transesophageal echocardiography (**C**)

TTE showed good graft function without any relevant abnormalities. Histological analysis of the EMB showed no rejection. Nor chest x-ray, neither abdominal sonography showed any relevant abnormalities. To identify the source of inflammation, a chest and abdominal computed tomographic (CT) scan was performed. This revealed a large, irregular, saccular dilatation of the tubular portion of ascending aorta with a maximum diameter of 90 mm (Fig. 1a, b).

### Therapeutic intervention

**Fig. 2:**
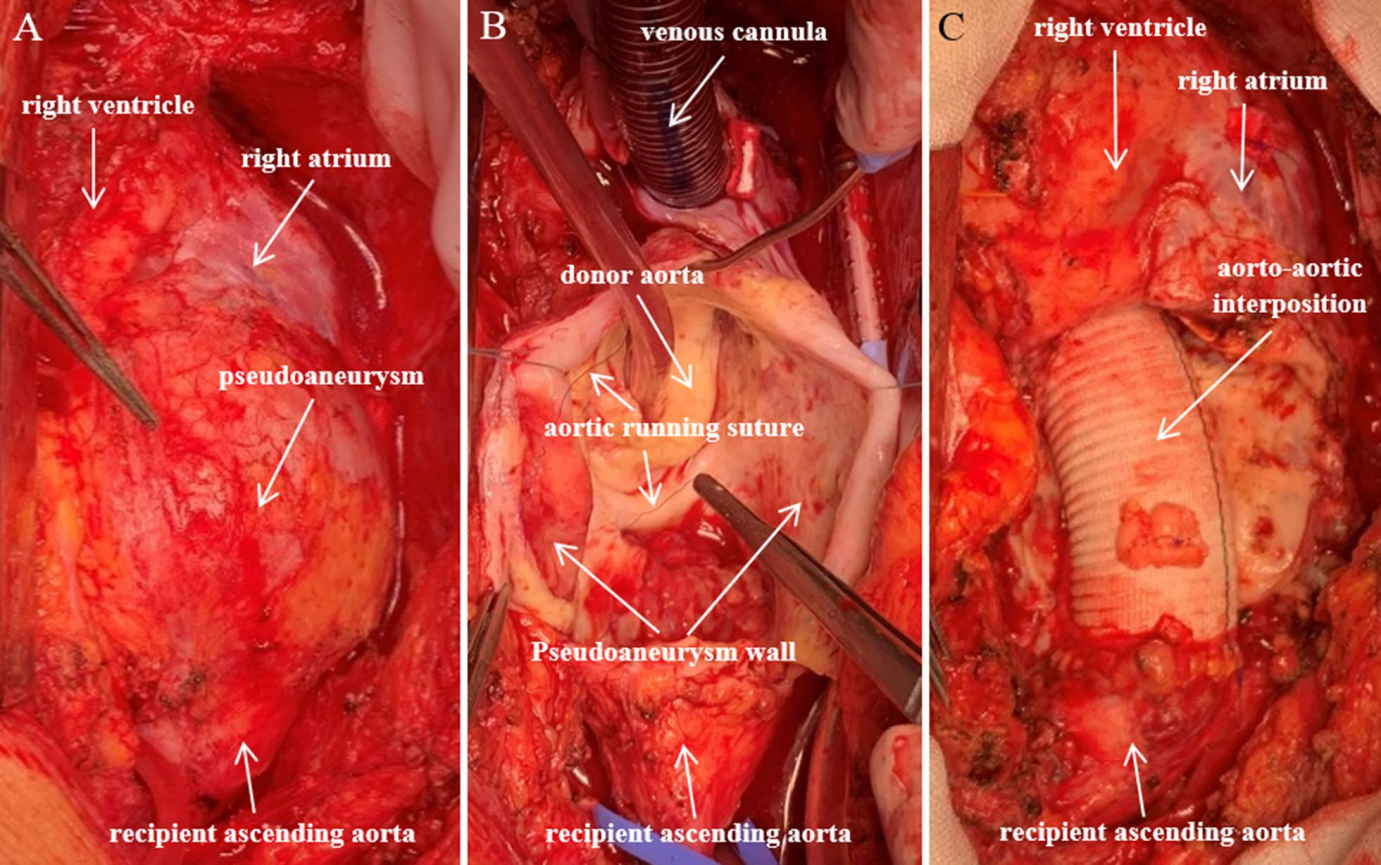
Intraoperative photographs of the ascending aorta. Images of the ascending aorta before and after its reconstruction. The aortic pseudoaneurysm was found to be intact after sternotomy (**A**). After opening it, rupture of the former running suture between the donor and the recipient aorta was detected (**B**). The pseudoaneurysm was resected and an aorto-aortic interposition was performed (**C**)

Based on chest CT findings, an urgent surgical intervention was performed on the following day. Before performing median resternotomy, the left femoral artery was canulated through an 8 mm dacron vascular prosthesis for arterial line of cardiopulmonary bypass (CPB), to ensure the safety of patient in case of a pseudoaneurysm rupture. Myocardium protection was provided by antegrade Custodiol cardioplegia. After aortic crossclamp, the aortic pseudoaneurysm (Fig. 2a) was opened. We identified the rupture of the former aortic polipropilene running suture (Fig. 2b), which could be the basis for the pseudoaneurysm formation between the donor and the recipient aorta. The tissue quality of both donor and recipient aorta seemed to be normal macroscopically. However, the tissues where the pseudoaneurysm wall attached to the donor aortic wall contained purulent-like debris, this was sent for microbiology examination. We resected the pseudoaneurym thoroughly and performed an aorto-aortic interposition between the recipient’s distal ascendent aorta and donor’s sinotubular junction with a 28 mm dacron vascular prosthesis (Fig. 2c). CPB time was 78 min, aortic cross clamp time was 48 min.

The patient recovered from surgery uneventfully. TTE showed unchanged graft function. No decline in renal function was detected.

Histologic evaluation of the resected pseudoaneurysm and aortic walls of the recipient and donor showed infectious aortitis, but no pathogenes could be identified. However, cultures of the resected specimens grew multisensitive *P. aeruginosa*. Serial blood cultures and urine culture were repeatedly negative.

The patient received 18 days of intravenous treatment of ceftazidime (2000 mg three times daily) followed by 7 months of oral ciprofloxacin (500 mg twice daily) from the day of discharge. The targeted antibiotic therapy resulted in significant decrease in CRP level.

In the early postoperative period mycophenolic acid therapy was transiently stopped and reinitiated in a reduced dose when inflammatory markers decreased. Tacrolimus and steroid regimen was maintained.

### Follow-up and outcome

At follow-up examinations the patient was doing well without fever, TTE-s showed no abnormalities and CRP level remained normal. Blood pressure could be kept in target range. EMB-s showed no sign of rejection.

**Fig. 3:**
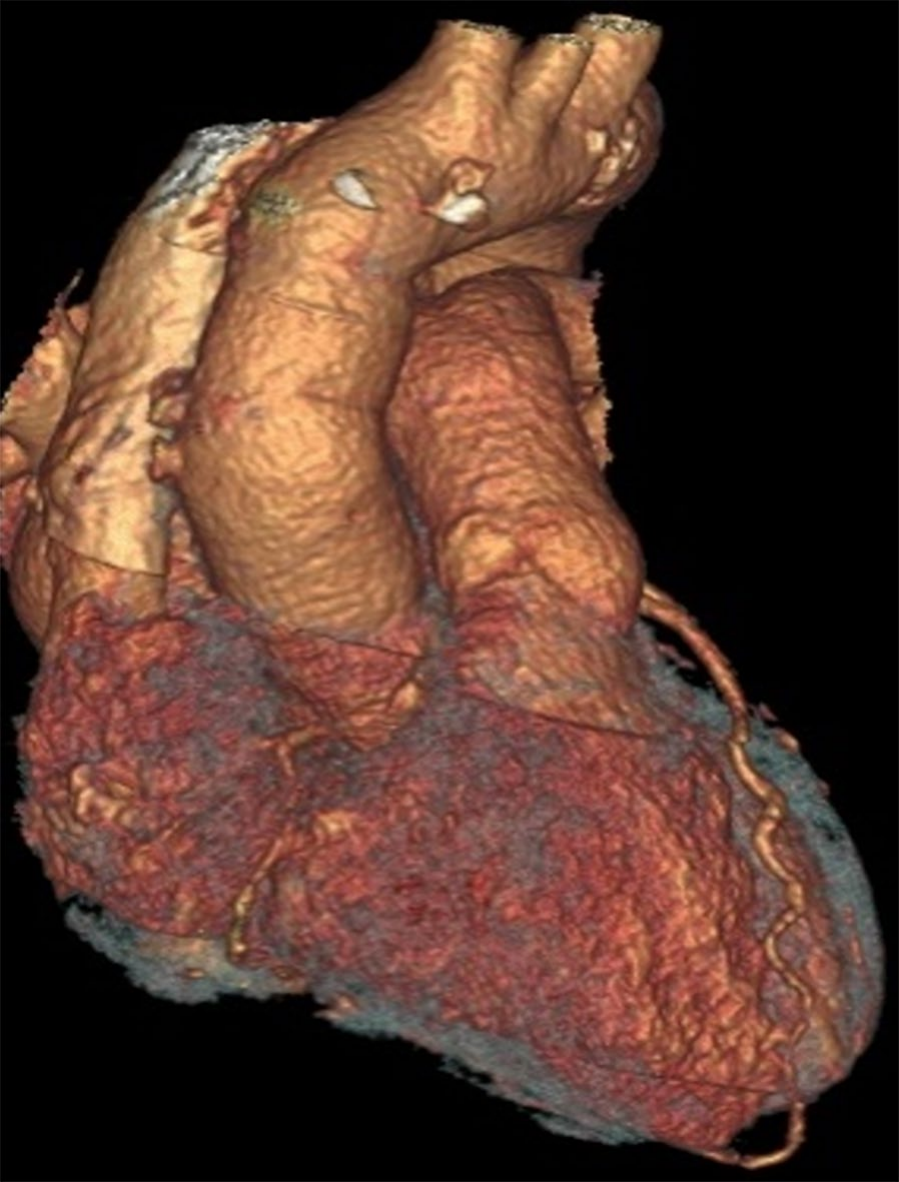
Three-dimensional CT image of the postoperative state of the ascending aorta. Three-dimensional CT reconstruction shows an intact interposition graft of the ascending aorta 7.5 months after the surgery

19 days and 7.5 months after the surgery chest CT angiograms showed intact interposition graft of the ascending aorta with no sign of recurrent aortic pseudoaneurysm (Fig. 3). Based on this finding and the continuously normal inflammatory markers of the patient, we decided to discontinue the oral ciprofloxacin therapy almost 8 months after the operation.

At his latest follow-up one year after the operation the patient is still asymptomatic with normal inflammatory markers.

## Discussion and conclusions

Mycotic pseudoaneurysms of the ascending aorta are rare complications after cardiac surgery and are caused by growth of microorganisms in the vessel wall. The name mycotic aneurysm was coined by Osler to describe aneurysms associated with bacterial endocarditis [3]. These were noted to have the appearance of "fresh fungus vegetations", however, the majority of mycotic aneurysms are caused by bacteria. Immunosuppression is a major predisposing factor for this complication.

**Fig. 4:**
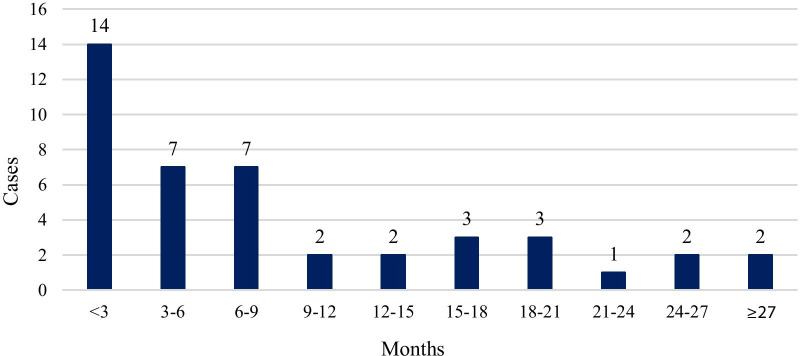
Time between heart transplantation and detection of pseudoaneurysm. Based on literature data, majority of the ascending aortic mycotic pseudoaneurysms are detected less than three months after heart transplantation. After nine months, number of newly diagnosed cases decreases markedly (49 cases reviewed, missing data in 6 cases)

After thorough review of literature we identified 49 case reports of mycotic pseudoaneurysms of the ascending aorta after HTX. Median time between transplantation and diagnosis of pseudoaneurysm was 6 months (Fig. 4). Resected specimen of ascending aortic pseudoaneurysms of heart transplant patients frequently grows *Staphylococcus aureus* [4–17], *P. aeruginosa* [7, 14, 18–23] or Candida species, [7, 14, 21, 24–29] but other gram-positive [7, 14, 30, 31] or gram-negative [14, 32] bacteria and *Aspergillus fumigatus* [33–35] are also reported as potential pathogens.

The following predisposing factors for aortic wall infection and formation of mycotic pseudoaneurysm are documented in literature: intrathoracic infection (50%) [6–8, 10, 14–16, 24, 25, 30, 33, 35, 36], pneumonia or bronchitis (20%) [4, 13, 17, 19, 21, 25, 26], heart–lung transplantation (17.5%) [4, 14, 15, 19, 25], reoperation due to bleeding (15%), [4, 8, 13, 31] diabetes mellitus (15%), [7, 12, 15, 31, 35, 37] infection of a previous ventricular assist device (12.5%) [9, 14, 22, 23], transmission of microorganisms from the donor (7.5%) [8, 25], acute cholecystitis (5%) [28, 33], urinary tract infection (5%) [18, 31], other septicaemia (5%), [17, 21] pacemaker endocarditis (2.5%), [13] skin disruption (2.5%), [29] heart–lung retransplantation (2.5%) [25].

In our patient there was no evidence of any obvious concomitant infection. However epiglottic abscess and Pseudomonas pneumonia of the donor is notable. Although both the donor and recipient received broad-spectrum antibiotic treatment, donor-derived Pseudomonas infection is possible as transport fluid of the donated right kidney also grew *P. aeruginosa*. *P. aeruginosa* grown from donor and recipient samples equally showed sensitivity for all antibiotics tested, which means they possessed a similar phenotype. Furthermore, the donor received only 24 h of adequate antibiotic therapy against Pseudomonas (imipenem/cilastatin) before the donation which might not have been long enough. These observations support the theory of donor-derived infection further. All available cases of Pseudomonas mycotic pseudoaneurysm in heart transplant recipients is summarized in Table 1. It is noteworthy that donor-derived Pseudomonas mycotic pseudoaneurysm in a heart transplant recipient has never been described before.

**Table 1:** Cases of ascending aortic mycotic pseudoaneurysm formed due to *Pseudomonas aeruginosa* infection after HTX

Age (years)	Sex	Time after HTX (months)	Recent verified infection or events predisposing to infection	Antimicrobial therapy	Surgical treatment	Follow-up (months)	Clinical outcome	Reference
39	Male	5	*Pseudomonas aeruginosa* septicaemia and positive urine culture	iv. Tobramycin, ceftazidime (6 weeks)	Resection, end-to-end anastomosis	8	unrelated death	Palac RT, et al. 1991
26	Male	15	Cystic fibrosis, heart–lung transplantation, *Pseudomonas aeruginosa* bronchitis	iv. Unspecified antibiotics (61 days) po. ciprofloxacin (continuously)	Direct repair (autologous pericardium)	5	operation due to recurrence, (resection of pseudoaneurysm and heterologous graft implantation), then unrelated death after 7 months	Cassart M, et al. 1994
31	Male	2	N/A	N/A	Resection, allograft implantation	N/A	reoperation due to recurrence, then recovered	McGiffin DC, et al. 1994
60	Male	N/A	Diabetes mellitus, mediastinitis	iv. Unspecified antibiotics (6 weeks)	Repair with allograft patch	34	recovered	Knosalla C, et al. 1996
N/A	N/A	8	*Pseudomonas pneumonia*	Unspecified antibiotics (4–6 weeks)	Collagen-sealed vascular graft implantation	2	reoperation due to recurrence, then death due to bleeding through bronchial fistula	Koyanagi T, et al. 1999
57	Male	29	Infected retained LVAD driveline (*Pseudomonas aeruginosa*)	iv. Unspecified antibiotics (≥ 6 weeks)	Resection, prosthetic graft implantation, pectoralis flap	1	recovered	Tang GHL, et al. 2011
44	Male	1.4	RVAD outflow graft infection, septicaemia (*Pseudomonas aeruginosa*)	Unspecified antibiotics	After 3 weeks: resection, allograft implantation	N/A	N/A	Mirzaee S, et al. 2016
45	Male	1.1	Pseudomonas isolated from previous LVAD exit site	iv. Ceftolozane/tazobactam (8 weeks) po. ciprofloxacin (lifelong)	Resection, allograft implantation	8,3	recovered	Aye C, et al. 2017

Eight cases of ascending aortic mycotic pseudoaneurysm formed due to *P. aeruginosa* infection after HTX were found in the literature. All of them were male patients with a median age of 44 years. The median time between HTX and diagnosis was 5 month. Ventricular assist device related infection was common in medical history. Combined surgical and antibiotic treatment was performed, which however resulted in a recurrence rate of 43%. N/A: not available

Possible symptoms of mycotic aortic pseudoaneurysms are malaise [4, 18, 22, 28], chest [6, 8, 10, 12, 28, 32] or back pain [6, 19, 23, 33], dyspnea [17, 25, 27, 30], hoarseness [21, 30] or fever [4, 6, 8, 9, 12, 13, 17–19, 22, 23, 27, 31–33]. However, completely asymptomatic cases are also reported [25, 26, 34, 37].

The patient demonstrated in the current case was almost fully asymptomatic except of an unexplainable weight loss. Weight loss as an usual general symptom of aortic pseudoaneurysm is reported in the literature as well and may be caused by the inflammatory state [11].

In the case of aortic pseudoaneurysm, common laboratory findings—also seen in our patient—are elevated serum CRP level and elevated white cell count, predominantly neutrophils [6, 23, 28, 37]. Highly elevated erythrocyte sedimentation rate is also documented as an alarming sign [37].

**Fig. 5:**
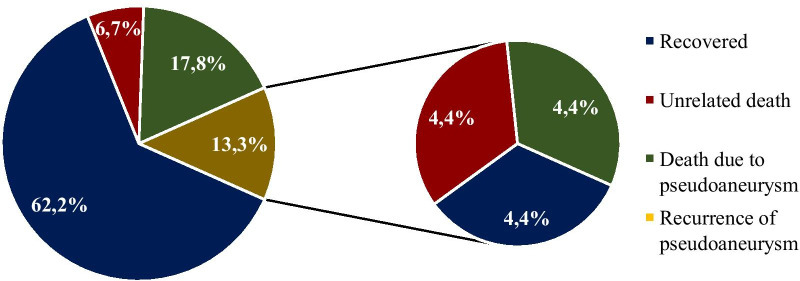
Outcomes of heart transplant patients diagnosed with ascending aortic mycotic pseudoaneurysms. Based on literature review, almost two-thirds of heart transplant patients recovered after treatment of their ascending aortic mycotic pseudoaneurysms. A sixth of patients died due to the pseudoaneurysm itself, an eighth of them suffered recurrence, while one fifteenth of patients died due to an unrelated cause. Among patients with recurrent pseudoaneurysms, ratio of recovery, pseudoaneurysm-related death and unrelated death was equal (49 cases reviewed, missing data in 4 cases)

Early diagnosis, surgical intervention and adjuvant antibiotic therapy seem to be the keys to successful management of mycotic pseudoaneurysms after HTX. Based on literature data of heart transplant patients with ascending aortic mycotic pseudoaneurysms with a median follow-up time of 8 months after diagnosis, almost two-thirds of patients recovered after operation [4–7, 9, 10, 13–15, 17, 21, 23, 24, 26–32, 34, 35, 37], a sixth of them died due to pseudoaneurysm [4, 6, 8, 14, 16, 25, 33], and an eighth of them presented with a recurrent pseudoaneurysm later [7, 8, 12, 19–21]. Median time between first surgical treatment for pseudoaneurysm and the verification of recurrence was 2 months. At the remaining cases (one fifteenth of patients), death due to an unrelated cause occured [8, 14, 18]. One-third of patients treated due to recurrent pseudoaneurysm recovered [12, 20], one-third of them died due to pseudoaneurysm [8, 21], and one third died due to other reason [7, 19] (Fig. 5).

It is obvious that the choice of antibiotic should be based on the culture and sensitivity of the isolated organism. Nevertheless, duration of intravenous and oral antibiotics in the case of *P. aeruginosa* aortitis after heart transplantation varies [7, 14, 18, 19, 21–23] and permanent antibiotic therapy is recommended by some relevant literature datas [19, 23]. Our strategy was the administration of targeted parenteral antibiotic treatment followed by continuous oral ciprofloxacin therapy. Systemic immunosuppression and synthetic aortic graft material are potential risk factors for recurrent graft infection, therefore, it is worth considering lifelong antibiotic treatment. However, it is also important to take care of potential side effects of permanent ciprofloxacin therapy including an increased risk of aortic aneurysm or dissection [38, 39].

In conclusion, we presented a patient in whom a *P. aeruginosa* pseudoaneurysm of the ascending aorta developed after HTX. In this case no obvious concomitant infection was found at the patient and donor-derived infection is suspected. This case demonstrates the importance of considering a mycotic pseudoaneurysm of the aorta in the etiology of unexplainable inflammation following HTX, even if the patient is almost asymptomatic. Surgical intervention and targeted antibiotic treatment are equally essential. *P. aeruginosa* as a causative organism may require long-term antibiotic therapy.

## Data Availability

All data supporting the conclusions of this article are included within the article.

## Abbreviations

CPB: Cardiopulmonary bypass
CRP: C-reactive protein
CT: Computed tomography
EMB: Endomyocardial biopsy
HTX: Heart transplantation
min: Minute
N/A: Not available
*P. aeruginosa*: *Pseudomonas aeruginosa*
TTE: Transthoracic echocardiography
